# Neural Networks for Estimating Speculative Attacks Models

**DOI:** 10.3390/e23010106

**Published:** 2021-01-13

**Authors:** David Alaminos, Fernando Aguilar-Vijande, José Ramón Sánchez-Serrano

**Affiliations:** 1 Department of Financial Management, Universidad Pontificia Comillas, 28015 Madrid, Spain; 2 PhD in Economics and Business, Universidad de Málaga, 29071 Málaga, Spain; fernando.aguilar@uma.es; 3 Department of Finance and Accounting, Universidad de Málaga, 29071 Málaga, Spain; joseramonsanchez@uma.es; 4 Cátedra de Economía y Finanzas Sostenibles, Universidad de Málaga, 29071 Málaga, Spain

**Keywords:** speculative attacks, currency crisis, neural networks, deep learning, Quantum-Inspired Neural Network

## Abstract

Currency crises have been analyzed and modeled over the last few decades. These currency crises develop mainly due to a balance of payments crisis, and in many cases, these crises lead to speculative attacks against the price of the currency. Despite the popularity of these models, they are currently shown as models with low estimation precision. In the present study, estimates are made with first- and second-generation speculative attack models using neural network methods. The results conclude that the Quantum-Inspired Neural Network and Deep Neural Decision Trees methodologies are shown to be the most accurate, with results around 90% accuracy. These results exceed the estimates made with Ordinary Least Squares, the usual estimation method for speculative attack models. In addition, the time required for the estimation is less for neural network methods than for Ordinary Least Squares. These results can be of great importance for public and financial institutions when anticipating speculative pressures on currencies that are in price crisis in the markets.

## 1. Introduction

A currency crisis is defined as the inability of the authorities of a country to defend a certain parity for the exchange rate. In turn, the exchange rate crisis will occur as a result of a speculative attack carried out by operators in the foreign exchange market, which causes a large and sudden increase in the ability to readjust the central parity [1]. The models of speculative attacks best known from the previous literature are the so-called first- and second-generation models. The first-generation models are based on the incompatibility between the economic policy of a government and its commitments to a fixed exchange rate, which ends up leading to a speculative attack on its currency and the collapse of the exchange regime. The first formulation of this type of model is due to Krugman [2]; second-generation ones incorporate private agents, their expectations, and interaction with economic policy, generating the possibility of multiple equilibria and self-generated crises. This second-generation model was built by the work of Obstfeld [3]. The experience of countries with exchange rate crises shows that they cause significant welfare losses for economic agents, insofar as they have generated falls in output and employment, and large losses in international reserves without neglecting significant fiscal problems. Hence the importance of having indicators that warn about events of excessive fragility is that they allow the authorities to act promptly to minimize the costs associated with the outcome of these episodes of speculative attacks in currency crises.

In the last decade, many countries have suffered a currency crisis that has led to high pressure against the price of their currency in financial markets [4]. This has been due to the significant deterioration of their balance of payments concerning international trade. However, the reasons why they have suffered these falls have been varied. Countries like Russia and Iran suffered in recent years different important falls in the value of their currency due to the economic sanctions imposed by the United States and the European Union. This caused a drop in their commercial activity, and therefore, an abrupt deterioration in their international trade balances. Other African countries such as Namibia or South Africa have also recently suffered acute currency crises due to domestic political crises and continuing instability that has deteriorated their international image and their bilateral and trade relations with other countries. Lastly, Latin American countries such as Mexico or Argentina have suffered successive currency crises with consequences of speculative attacks due to their current account crises with failed economic policies.

Different authors have analyzed speculative attacks based on macroeconomic theory, being the object of continuous study and with strong consequences both in the economy and in the financial markets. However, in the last decade, we can find various works on speculative attacks with very specific objectives on the procedure in which they occur. Even so, these studies have not obtained a great repercussion, the first- and second-generation models created previously are currently of great importance [5,6,7,8,9,10]. Others that follow this line of speculative attack models stand out, such as those carried out by [11,12,13,14,15,16,17], where they have tried to explain the origins of speculative attacks and currency crises, managing to establish the theory that helps to explain these phenomena. This has also been studied in various works such as those of [15,16,17,18,19,20,21] discussing what type of exchange rate to establish or what type of economic policy to choose to reduce the chances of suffering a speculative attack. Despite this, recent previous literature has revealed difficulties in achieving a certain degree of predictive capacity [15,16,17,21]. The current complexity in economic decisions and especially in financial markets leads to the need to search for new methodologies that more accurately estimate the models of speculative attacks. These models on speculative attacks have always been estimated using the Ordinary Least Squares (OLS) method, as the most widely used statistical technique in estimating these models [7,8,9,10,11,12,13,14,15].

In order to cover this gap, and given the importance that currency trading problems continue to have for many countries, the present study develops different machine learning techniques for estimating the two main popular speculative attacks models that respond to the most current concerns of the financial situation of the currencies. To this end, the data have been used for the cases of Mexico and Thailand, two countries that in recent decades have shown difficulties with the price of their currencies, being targets of attacks by numerous agents in the foreign exchange market. Specifically, the neural networks of Perceptron Multilayer, Deep Recurrent Neural Networks, Deep Neural Decision Trees, and Quantum-Inspired Neural Networks have been used, to be compared with the usual OLS method. The quantum variant is the one that achieves the best results both outside the sample and also in the forecasts of final postestimations made. Besides, the computational methodologies used in this study improve the precision results obtained by the OLS method. These results are repeated for both the first-generation and second-generation models, as well as for the data used from Mexico and Thailand.

We make some contributions to the literature. We consider new estimation techniques for forecasting the speculative attacks through the first- and second-generation models, testing the precision and level of residuals obtained by each methodology. It has important implications for public institutions, governments, central banks, financial institutions, and other stakeholders concerned in the foreign exchange markets for the accurate estimation of speculative attacks.

The present study is organized as follows: Section 2 reviews the speculative models used in this study. In Section 3, the methods used are presented. In Section 4, the data and the variables used in the research are detailed and the results obtained are analyzed. Finally, the conclusions of the study and its implications are exposed.

## 2. Speculative Attacks Models

### 2.1. First Generation Model

The models of currency crisis or balance of payments crisis try to explain why and the logic of how a currency crisis is unleashed. Thus, the first-generation models were based, mainly, on the fact that exchange rate crises occur due to the existence of incompatibility in monetary and fiscal policies (both expansive) with the maintenance of a fixed exchange rate regime in the long term. In other words, these occur in a situation in which a government (central bank), which promised to keep the exchange rate fixed, is running constant fiscal deficits and these are monetized by its central bank. This situation creates an incompatibility that will mean that this exchange rate regime cannot be maintained for long. The reason why this regime will end up collapsing is that there is a surplus of the money supply over demand continuously and this surplus will be reduced by the central bank by selling reserves. Thus, the central bank will lose reserves in all periods to balance the money market. Faced with this situation of constant loss of reserves, investors, anticipating the natural disappearance of reserves, will carry out a speculative attack on the local currency that will lead to reserves decreasing to a “critical” value, a level that may be zero according to the Flood and Garber model [10] or that they reach a level below the critical value [1,2,3].

The first-generation basic model considers that private agents (investors or speculators) have perfect foresight on the future behavior of economic variables and work in continuous time. It is a model that assumes a small and open economy, where a single good is produced, and it is assumed that the Purchasing Power Parity (PPP) and the discovered interest parity are met. There are two types of assets, local and foreign money, and bonds, also local and foreign, the latter perfectly substitutes (this implies the existence of an interest rate). The model proposes a small country, where it produces a marketable good in the international market, whose price in the national territory (*P*) is defined by the exchange rate (TC) of the national currency expressed in terms of the foreign currency (*s*) multiplied by the price of the product in international markets (*P* *), as it appears in expression (1),

*P* = *sP* *, (1)

The hypothesis also assumed that the price of the good abroad *P* * is constant and equal to 1 (*P* * = 1). So, the internal price of the product will be equal to the exchange rate (*P* = *s*).

The approach of Krugman is completed with flexible wages and prices, with production in full employment, and the trade balance, regardless of the role of the balance of payments in the current account model, will be the difference between production and expenditure:

*B* = *Y − G − C*(*Y − T*,*W*)   *C*_1_,*C*_2_ > 0, (2)

where *B* is the current account balance, *Y* is the level of production, *G* defines public spending, *C* represents private consumption, *T* is the tax variable, and *W* is total household wealth.

Regarding the asset market, the model establishes that investors can only choose between two assets: national currency (*M*), and foreign currency (*F*), with the nominal interest rate of both assets equal to zero. In this way, the real wealth of national residents (*W*) will be equal to the sum of holdings in the national currency (*M*) plus those of foreign currency (*F*) as defined in expression (3):

(3) $$W=\frac{M}{P}+F.$$

Lastly, the model assumes that foreigners do not have a national currency, so (*M*) represents the national currency stock, and in equilibrium, it assumes that national residents must be willing to maintain said stock. The equilibrium condition of the portfolio establishes that asset holdings in national currency are equivalent to a proportion of residents’ real wealth and that this, in turn, depends on the expected inflation rate (*π*). Furthermore, one of the assumptions of the model is that the domestic price level (*P*) corresponds to the exchange rate (s), and asset holdings in national currency depend on the expected depreciation rate of the currency, expressed in Equation (4):

(4) $$\frac{M}{P}=L\left(\pi \right)\times W.$$

Krugman considers two different economic regimes: a system with a flexible exchange rate and a system with a fixed exchange rate. The behavior of the economy in the short term is different depending on the exchange rate system. An increase in the expected inflation rate under a flexible exchange rate regime produces an increase in the domestic price level, while when the exchange rate is fixed, an increase in the expected inflation rate implies an alteration in the composition of residents’ wealth, increasing foreign currency assets (∆*F*) and decreasing domestic currency assets. This situation causes a compensatory change in government reserves that decrease by the same amount as holdings of foreign currency in the hands of private residents increase:

(5) $$\Delta R=-\Delta F=\Delta \frac{M}{P}.$$

Krugman also analyzes the dynamic behavior of the economy under both exchange rates. In the case of flexible TC, it is assumed that the creation of money depends solely on the financing needs of the government. Therefore, the growth of the money stock will be determined by the differences between the government’s fiscal expenses and revenues, as expressed in Equation (6):

(6) $$\frac{M}{P}=G-T.$$

Relating public spending and money supply, under the assumption of perfect forecasting of the inflation rate, Krugman shows that the demand for assets in national currency will depend exclusively on price growth and that national residents will only be willing to increase the proportion of national currency over foreign currency if there is a reduction in the price level.

In a fixed exchange rate regime, it is assumed that the government has a stock of reserves in foreign currency, which it uses to stabilize the exchange rate. This is equivalent to saying that the price level is constant, where *P* = *sP* * and *P* * = 1, and therefore *P* = *s* = 0. The private sector can only acquire assets if it decreases its spending relative to its income and therefore, private sector savings are considered:

*S* = *Y* − *T* − *C*(*Y* − *T*,*W*). (7)

In this case, and because the price level is constant, the growth of residents’ wealth is equivalent to the savings of the private sector, that is:

(8) $$\dot{W}=\frac{\dot{M}}{\bar{P}}+\dot{F}=S.$$

In this way, the distribution of savings between assets denominated in national currency and assets in a foreign currency will be determined by the equilibrium condition of the trade balance. As long as investors trust the government to maintain the price level, the expected inflation will be zero, giving a stable relationship between wealth and deposits in national currency. If there is an increase in the wealth of residents, a proportion *L* will go to the national currency, given: $\frac{M}{P}=L\left(\pi \right)\times W$ and (1 − *L*). It will be used for assets in foreign currency. The government will be able to cover its deficit by issuing new national currency or by using its foreign currency reserves (*R*). Therefore, the composition of the state budget can be expressed:

(9) $$\frac{\dot{M}}{\dot{P}}+\dot{R}=G-T=g\left(\frac{M}{P}\right).$$

From this expression, it follows that if the government commits to maintaining the exchange rate, it has no control over how it finances its deficit. Over time, both private sector wealth and government reserves will vary. When the government runs a deficit, its reserves decrease, even though the private sector saving is zero. In a deficit situation, fixing the exchange rate is impossible regardless of the initial amount of reserves that the government had and the effect derived from said fixing will generate a balance of payments crisis, caused by a speculative attack at the moment in which the agents anticipate the depletion of reserves.

### 2.2. Second Generation Model

The second-generation models differ from the first generation because they are models of multiple equilibria, since they consider an interaction between the private sector and the behavior of the government, giving rise to multiple solutions. These second-generation models consider that in a country’s economy, there is an interrelation between the behavior of the private sector and the decisions made by the public sector. Thus, a financial crisis under this relationship can take place when international financial operators have expectations about a possible devaluation of the currency, this situation is reflected in interest rates, which by rising try to attract national currency against the foreign currency. This scenario can lead the government to devalue due to the cost of debt service. On the contrary, if the private agents do not have expectations that the exchange rate will change, the interest rate remains low and the devaluation is less likely.

Second-generation models were developed by Flood and Marion [11] to understand crises in their self-fulfilling character. According to this mechanism, if the agents foresee a possible devaluation of the currency, this will be reflected in the salary negotiations, which will cause economic imbalances, including a rise in the country’s price level. These imbalances can be corrected by the government through the exchange rate since it is set after wage negotiations. If the government decides not to devalue, it will correct economic imbalances avoiding an increase in inflation by reducing its control over the variables that define the level of production. If, on the contrary, the government decides to lean towards the flexible exchange rate, it will be feeding a process through which both the level of wages and prices in the country will increase. Both situations are reflected in Equation (10), which reflects the so-called cost of the exchange rate regime.

(10) $$L_{t}=0.5\theta \left(p_{t}-p_{t-1}\right)+0.5{\left(y_{t}-y^{*}\right)}^{2},$$

where *p_t_* is the national price level, *y_t_* is the country’s output at time *t*, y* is the output target set by economic policy, and *θ* is the weight associated with deviations in inflation from the political objective.

According to this approach, the government will decide to devalue its currency provided that the loss for leaving the fixed exchange rate system, together with the cost for the government of the loss of credibility of making this decision, is less than the loss obtained for not giving up under pressure and keep the exchange rate fixed. In this model, the existence of different levels of economic equilibrium stands out, where each level reflects the expectations that economic agents maintain about the economic policy that the government will carry out in the following period, since depending on the levels of devaluation expectations, the parameters of the equation will also be different, thus obtaining multiple results.

## 3. Neural Networks Methods

### 3.1. Multilayer Perceptron (MLP)

The multilayer perceptron (MLP) is a feed-forward, supervised artificial neural network model that is composed of a layer of input units, another layer of output, and several intermediate layers called hidden layers in so much so that they have no connections with the outside world. Each input sensor would relate to the units of the second layer, these in turn with those of the third layer, and so on. The network will aim to establish a correspondence between a set of input data and a set of desired outputs.

Moreover, [22] show that learning in MLP was a special case of a functional approach, where there is no assumption about the model underlying the data analyzed. This process involves finding a function that correctly represents the learning patterns, in addition to carrying out a generalization process that allows the efficient treatment of unanalyzed individuals during said learning. To do this, we proceed to adjust the W weights from the information from the sample set, considering that both the architecture and the network connections are known. The objective is to obtain those weights that minimize the learning error. Given, then, a set of pairs of learning patterns {(*x*_1_, *y*_1_), (*x*_2_, *y*_2_)… (*x_p_*, *y_p_*)}, and an error function *ε*(*W*, *X*, *Y*), the training process implies the search for the set of weights that minimizes the learning error *E* (*W*), as expressed in (11).

(11) Ewmin(W)= wmin∑i=1pε(W,xi,yi).

Most of the analytical models used to minimize the error function use methods that require the evaluation of the local gradient of the *E*(*W*) function and techniques based on second-order derivatives can also be considered [23,24].

### 3.2. Deep Recurrent Convolution Neural Network

Recurrent neural networks (RNN) have been successfully used in many fields for time-series prediction due to its huge prediction performance. For a simple neural network (NN), the inputs are assumed to be independent of each other. The common structure of RNN is organized by the output of which is depended on its previous computations [24,25]. Given an input sequence vector *x*, the hidden states of a recurrent layer *s*, and the output of a single hidden layer *y*, it can be calculated as appears in expressions (12) and (13):

(12) $$s_{t}=\sigma \left(W_{xs}x_{t}+W_{ss}s_{t-1}+b_{s}\right)$$

(13) $$y_{t}=ο\left(W_{so}s_{t}+b_{y}\right)$$

where *W_xs_*, *W_ss_*, and *W_so_* denote the weights from the input layer *x* to the hidden layer *s*, the hidden layer to itself, and the hidden layer to its output layer, respectively. *b_s_* and *b_y_* are the biases of hidden layer and output layer, respectively. *σ* and *o* are the activation functions. The Equation (14) represents the function of vibration signals.

(14) $$STFT\left\{z\left(t\right)\right\}\left(\tau ,\omega \right)\equiv T\left(\tau ,\omega \right)=\int_{-\infty \;}^{+\infty \;}z\left(t\right)\omega \left(t-\tau \right)e^{-j\omega t}\;dt$$

where *z* (*t*) is the vibration signals, *ω* (*t*) is the Gaussian window function focused around 0, and *T* (*τ*, *ω*) is a complex function that describes the vibration signals over time and frequency.

When time-frequency features {*T_i_*} are used to estimate speculative attacks with RNN, the convolutional operation is conducted in the state transition. To calculate the hidden layers with a convolutional operation, the next Equations (15) and (16) are applied:

(15) $$S_{t}=\sigma \left(W_{TS}\times T_{t}+W_{ss}\times S_{t-1}+B_{s}\right)$$

(16) $$Y_{t}=o\left(W_{YS}\times S_{t}+B_{y}\right)$$

where the term *W* indicates the convolution kernels. The convolutional operation has been determined by local connections, weight sharing, and local grouping, which allow every unit to integrate time-frequency data in the current layer. The convolution is operated between weights and inputs and is performed in the transition of inputs to the hidden layers.

Recurrent Convolutional Neural Network (RCNN) can be heaped to establish a deep architecture, named “deep recurrent convolutional neural network” [25]. When DRCNN is used to estimate speculative attacks, the last part of the model is a supervised learning layer, which is determined as appears in Equation (17):

(17) $$\hat{r}=\sigma \left(W_{h}\times h+b_{h}\right)$$

where *W_h_* is the weight and *b_h_* is the bias. The error between predicted observations and actual ones in the training data for speculative attacks estimation can be calculated and back propagated to train the model [25]. Considering that the actual data at time *t* is *r*, the loss function is determined as shown in the next Equation (18):

(18) $$L\left(r,\hat{r}\right)=\frac{1}{2}∥r-\hat{r}{∥}_{2}^{2}$$

Stochastic gradient descent is applied for optimization to learn the parameters. The gradient of loss function regarding parameters *W_h_* and *b_h_* are determined as follows in the Equations (19) and (20):

(19) $$\frac{\partial L}{\partial W_{h}}=-\left(r-\hat{r}\right)\sigma^{\prime }\left(.\right)h$$

(20) $$\frac{\partial L}{\partial b_{h}}=-\left(r-\hat{r}\right)\sigma^{\prime }\left(.\right)$$

### 3.3. Deep Neural Decision Trees (DNDT)

DNDT are DT models executed by deep-learning NNs, where a configuration of DNDT weightings corresponds to a specific decision tree and is thus interpretable [26]. The algorithm begins by implementing a soft binning function [27,28,29] to calculate the error rate for each node, making it possible to make decisions divided into DNDT. In general, the input of a binning function is a real scalar x, which generates an index of the containers to which x belongs. Assuming x is a continuous variable, group it into n + 1 intervals. This requires n cut-off points, which are trainable variables in this context. The cut-off points are denoted as [β1, β2,…, βn] and are strictly ascending such that β1 < β2 <…< βn.

The activation function of the DNDT algorithm is implemented based on the NN defined in Equation (21).

π = fw,b,τ (x) = softmax((wx + b)/τ),(21)

where w is a constant with value w = [1, 2,…, n + 1], τ > 0 is a temperature factor, and b is defined in Equation (22).

b = [0, − β1, − β1 − β2,…, −β1 − β2 − · · · − βn](22)

The NN defined in Equation (22) gives a coding of the binning function x. Additionally, if τ tends to 0 (often the most common case), the vector sampling is implemented using the Straight-Through (ST) Gumbel–Softmax method [30].

Given the binning function described above, the key idea is to build the DT using the Kronecker product, assuming we have an input instance x *∈ R^D^* with *D* characteristics. Associating each characteristic x_d_ with its own NN f_d_ (x_d_), we can determine all the final nodes of the DT, in line with Equation (23).

z = f1(x1) ⊗ f2(x2) ⊗···⊗ f_d_(x_d_) (23)

where z is now also a vector that indicates the index of the leaf node reached by instance x. Finally, we assume that a linear classifier on each leaf z classifies the instances that reach it.

However, the main drawback of the design is the use of the Kronecker product, which means it is not scalable in terms of the number of characteristics. In our current implementation, we avoid this problem using broad datasets and training a forest with random subspace [27,28,29,30]. This involves introducing multiple trees and training each with a subset with random characteristics. A better solution that does not require a forest of hard interpretability involves exploiting the dispersion of the binning function during the learning, since the number of nonempty leaves grows much slower than the total.

### 3.4. Quantum-Inspired Neural Networks (QNN)

The QNN is built from quantum computation techniques. These neural networks are inspired in quantum framework. The calculation unit of this model consists of quantum gates and their inputs and outputs are qubits. Any gate can calculate any local unit operation on the inputs. Quantum gates are interconnected by links. A quantum computational network is a computing machine that consists of quantum gates with synchronized steps. The calculation is done from left to right. The outputs of the gates are connected to the inputs of others. Some of the inputs are used as input to the network. Other inputs are connected to gates for 0 and 1 qubits. A few outputs are connected to sink gates, where arriving qubits are rejected [31,32]. An output qubit can be measured across the state |0〉 and |1〉, and is watched based on the probability amplitudes associated with the qubit [33,34,35]. Qubit is defined as the smallest unit of information in quantum computation, which is a probabilistic representation. A qubit may either be in the “1” or “0” or in any superposition of the two [36]. The state of the qubit can be defined as follows in the Equation (24):

(24) |ψ〉=α|0〉+β|1〉,

where α and *β* are the numbers that point out the amplitude of the corresponding states such that $|\alpha {|}^{2}+|\beta {|}^{2}=1$. A qubit is defined as the smallest unit of information in quantum computation. It is determined as a pair of numbers $\left[\begin{array}{l} \alpha \\ \beta \end{array}\right]$. An angle θ is a specification that represents geometrical aspects and is defined such that: cos(θ)=|α| and sin(θ)=|β|. Quantum gates may be applied for adjusting the probabilities because of weight upgrading [31,37]. An example of rotation gate can be: expressed as appears in the expression (25):

(25) $$U(\Delta \theta )=\left[\begin{array}{l} \cos(\Delta \theta )\;-\sin(\Delta \theta )\\ \sin(\Delta \theta )\;\;\;\;\;\;\cos(\Delta \theta ) \end{array}\right]$$

A state of the qubit can be upgraded by applying the quantum gate explained previously. Application of rotation gate on a qubit is defined as follows in expression (26):

(26) $$\left[\begin{array}{l} \alpha^{\prime }\\ \beta^{\prime } \end{array}\right]=\left[\begin{array}{l} \cos(\Delta \theta )\;-\sin(\Delta \theta )\\ \sin(\Delta \theta )\;\;\;\;\;\;\cos(\Delta \theta ) \end{array}\right]\left[\begin{array}{l} \alpha \\ \beta \end{array}\right]$$

The next hybrid quantum-inspired neural network is proposed for forecasting speculative attacks. The process is begun with a quantum hidden neuron from the state |0〉. The superposition expressed in the Equation (27) is prepared:

(27) $$\sqrt{p}|0\rangle +\sqrt{1-p}|1\rangle \;with\;0\leq |p|\leq 1,$$

where *p* represents random probability of starting the system in the state |0〉. The classical neurons are initiated by random number generation. The output from the quantum neuron is determined as follows in the Equation (28):

(28) $$v_{j}=f\left(\sum_{i=1}^{n}w_{ji}\times x_{i}\right)$$

where *f* is a problem-dependent sigmoid or Gaussian function. The output from the network is represented as appears in the Equation (29):

(29) $$y_{k}=f\left(\sum_{j=1}^{l}w_{jk}\times v_{j}\right)$$

The desired output is the *o_k_*. The squared error (*E*^2^*_k_*) is defined in the expression (30):

(30) $$E^{2}{}_{k}=\frac{1}{2}|y_{k}-o_{k}{|}^{2}$$

The learning follows the rules of the feed forward backpropagation algorithm. The upgrading of output layer weight is defined as follows in the Equation (31):

(31) $$\Delta w_{jk}=\eta e_{k}f^{\prime }\;v_{j}$$

Upgrading of quantum hidden layer weight in quantum backpropagation algorithm, the weights are upgraded by quantum gate conforming to Equation (26), so in this case, the equation would be as it appears in the Equation (32):

(32) $$\left[\begin{array}{l} \alpha_{ij}{}^{\prime }\\ \beta_{ij}{}^{\prime } \end{array}\right]=\left[\begin{array}{l} \cos(\Delta \theta )\;-\sin(\Delta \theta )\\ \sin(\Delta \theta )\;\;\;\;\;\;\cos(\Delta \theta ) \end{array}\right]\left[\begin{array}{l} \alpha_{ij}\\ \beta_{ij} \end{array}\right]$$

where $\Delta \theta_{ij}=-\frac{\partial E}{\partial \theta_{ij}}$, the index *i* represents the number of outputs from quantum neuron and the index *j* defines the number of outputs from network, $\gamma_{ij}{}^{\prime }\;=\gamma_{ij}+\eta \Delta \theta_{ij}$, and η is the learning rate [36,37]. This ratio usually takes the value of 0.1.

## 4. Data and Variables

The present study employs a sample of the quotations of the Mexican peso (MXN) and the Thai baht (THB). There have been two cases of currencies that have suffered speculative attacks in the past and analyzed by previous literature [1,2,3]. The period analyzed includes from 1995 to 2019, with the quotations of the currencies mentioned concerning the US dollar. In addition, the macroeconomic data of the current account balance, gross domestic product (GDP), consumption, total household wealth, inflation rate, assets in foreign currency, national savings, public spending, tax revenues, foreign currency reserves, quotation of the Mexican peso, the Thai baht against the US dollar, etc. have been used. These data have been obtained from Yahoo Finance, Federal Reserve Economic Data of St. Louis (FRED), and Open Data World Bank.

Besides, to check the reliability level of the models built, different test samples were created. This sample data set has been divided into mutually exclusive two groups, i.e., one for training (70% of the data) and another for testing (30% of the data). As is well known, the training data are used to fit the parameters of the models. For its part, the testing data are used to evaluate the built model and make predictions. The percentage of correctly classified cases (accuracy) and the root of the mean square error have been used for the evaluation. Furthermore, for the treatment of each of the three groups, the 10-fold cross-validation procedure has been applied with 500 iterations [33]. On the other hand, for our estimations, we used two four-core Intel Core i7-6500 processor as computing resources to make estimates. The code for the estimation of our methods has been performed by Python (3.8 version), with the support of the libraries such as NumPy, PyTorch, and QisKit to create the mathematical routines, Deep Learning algorithms, and Quantum processing, respectively. The MLP and OLS models have been created with MATLAB code (MATLAB R2016b package).

## 5. Results

**Figure 1:**
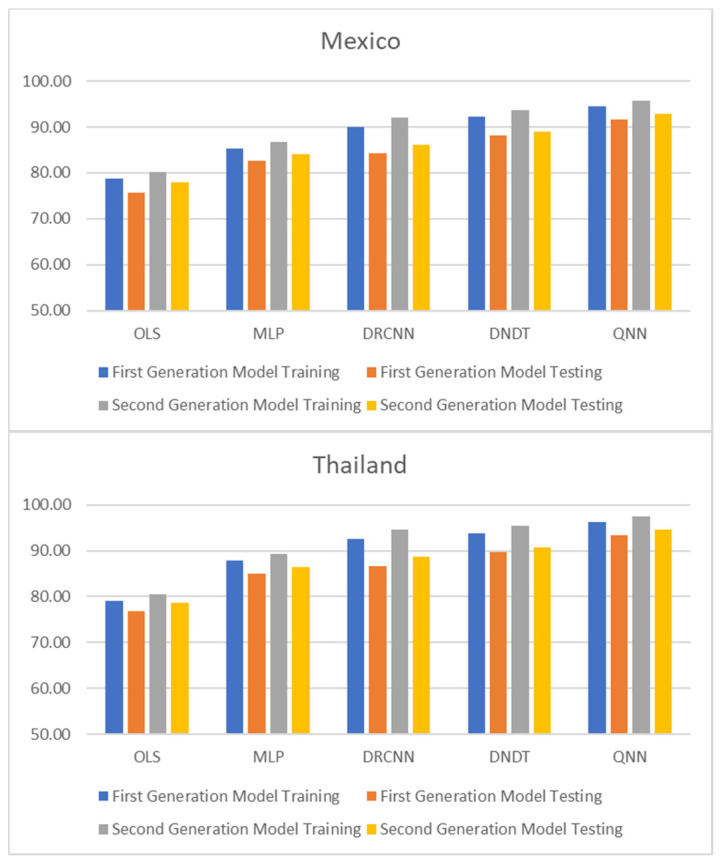
Results of accuracy evaluation: classification (%).

**Figure 2:**
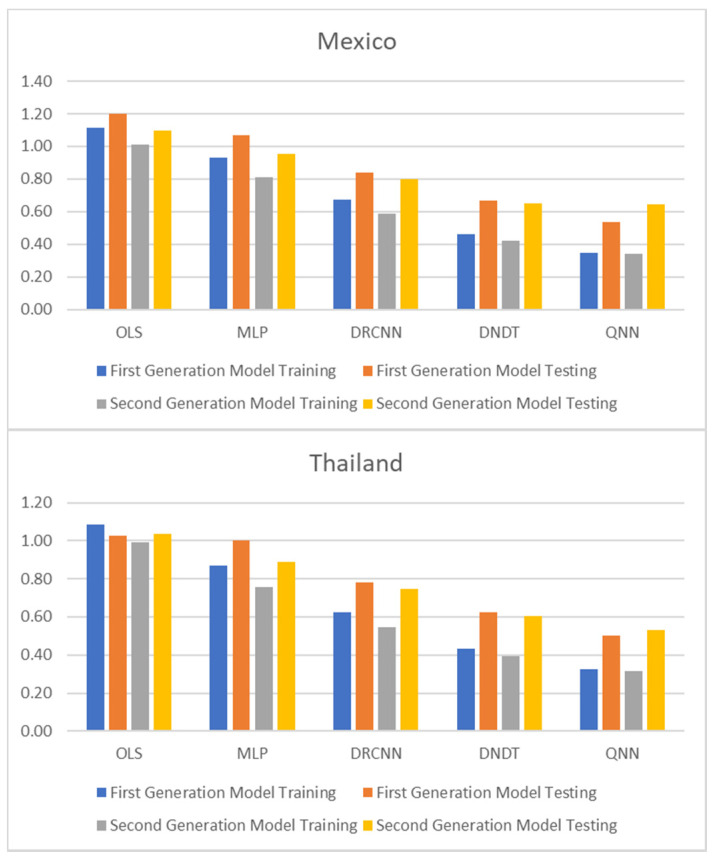
Results of accuracy evaluation: mean square error (RMSE).

**Figure 3:**
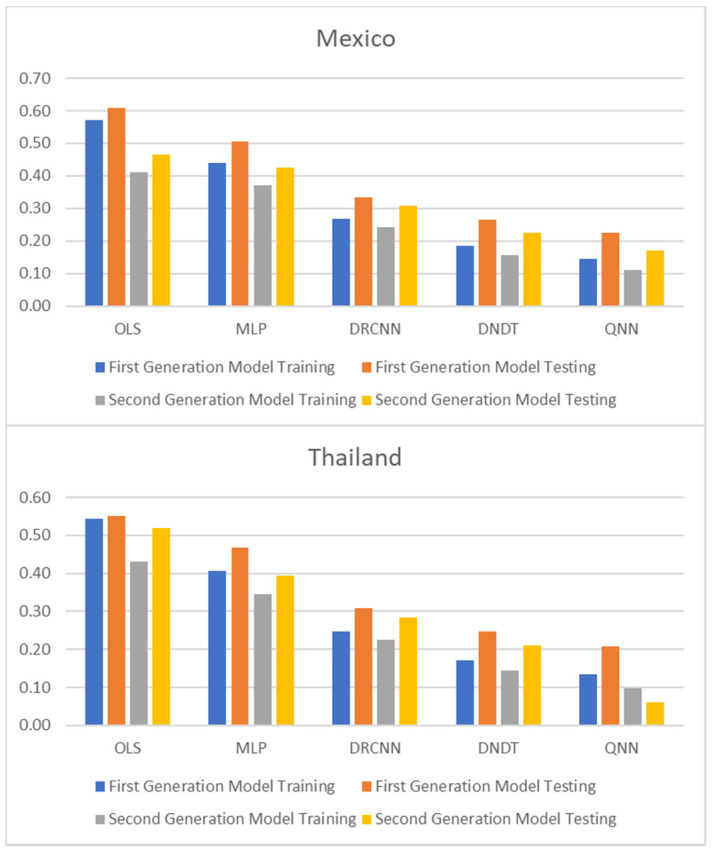
Results of accuracy evaluation: mean absolute percentage error (MAPE).

Table 1 and Table 2, and Figure 1, Figure 2 and Figure 3 show adjustment levels using accuracy, the mean square error (RMSE), and the mean absolute percentage error (MAPE). In all computational methods, the level of accuracy always exceeds 82.64% for testing data, while for OLS, it reaches 75.27% for Mexico and 77.41% for Thailand. For its part, the RMSE and MAPE levels are adequate. Therefore, computational methods improve OLS by a large margin, with QNN being the one that best adjusts the result in terms of residuals (with 91.62% accuracy), followed by DNDT (with 88.10%) for Mexico. In the case of Thailand, the results improve slightly, but the order of precision is the same since the best methodology is QNN with 92.84% in test data, followed by DNDT with 89.05%. Taken together, these results provide a level of accuracy far superior to that of previous studies. Thus, in the work of [7], an accuracy of around 78.2% is revealed. In the work of [9], it is close to 73.1%, and in the study of [12], it approaches 71%. Other studies such as [1,2,3,5,6] achieve a precision of even less than 70%. Therefore, the difference shown by the computational methodologies applied in this study far exceeds the precision shown by the previous literature.

These results demonstrate the greater stability offered by the QNN model compared to the rest, especially in the light of the RMSE and MAPE results obtained for three other computational methods. The results of the QNN improve the results of the popular OLS, just as it improves the precision results shown in previous works such as [9,10,11,12,13]. This set of computational methods observed as highly accurate represents a group of novel methods that estimate the speculative attacks and therefore different from that shown in the previous literature.

**Figure 4:**
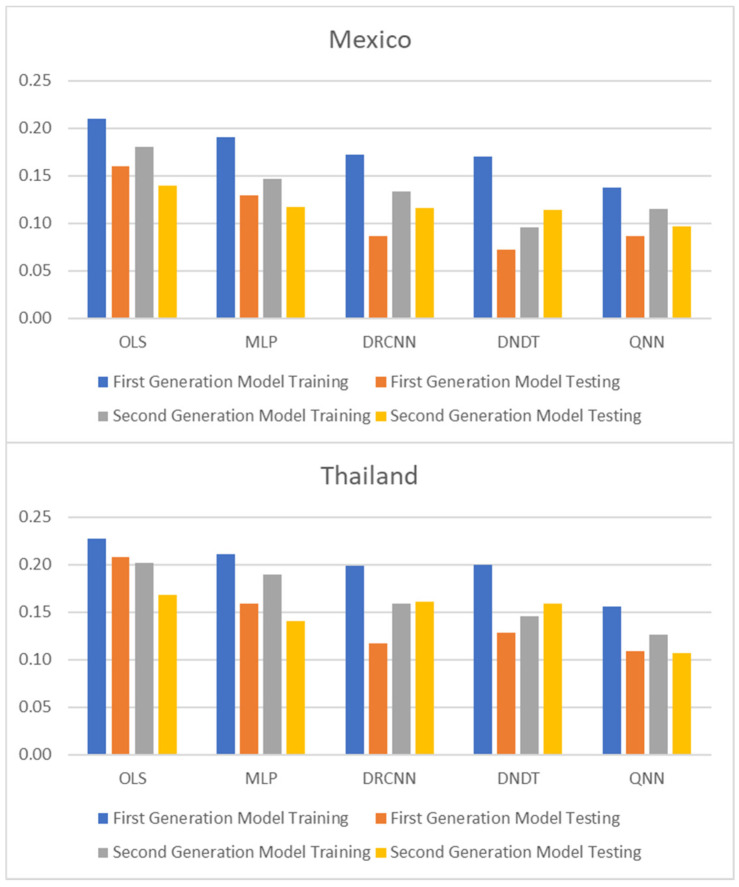
Results of time lapse for estimation.

To reinforce the superiority of neural network methodologies for estimating speculative attack models, the Diebold-Mariano (DM) and Harvey-Leybourne-Newbold (HLN) tests [38,39] have been applied to compare the methodologies used and the time elapsed to perform the estimation with each of the techniques. Table 3 reports the results of the DM test, showing that all the neural network methodologies used are better options than OLS. Like QNN, it is the best option compared to the rest, since the DM test ensures that the results that exceed 1.96/−1.96 do not reject the null hypothesis at 5% of significance, and therefore the differences observed between methodologies in the estimate are significant. On the same line, being the result with a negative sign means that the second option of the comparative is better than the second option. Likewise, the HLN test is adjusted version of DM test [39], which has better small-sample properties. Both DM and HLN tests show a significance difference between computational and statistical techniques, and the computational superiority over conventional methods. On the other hand, Figure 4 shows the average run time of the methodologies used for the estimation, where it is shown that neural network methodologies need a shorter estimation time, both for training and testing data, with QNN being the most common option efficient in terms of time use, needing 0.11 and 0.10 min to estimate with training and testing data, respectively, in the case of Mexico. For the case of Thailand, the estimate needs 0.13 and 0.11 min to estimate with training and testing data, respectively.

### Postestimations

**Figure 5:**
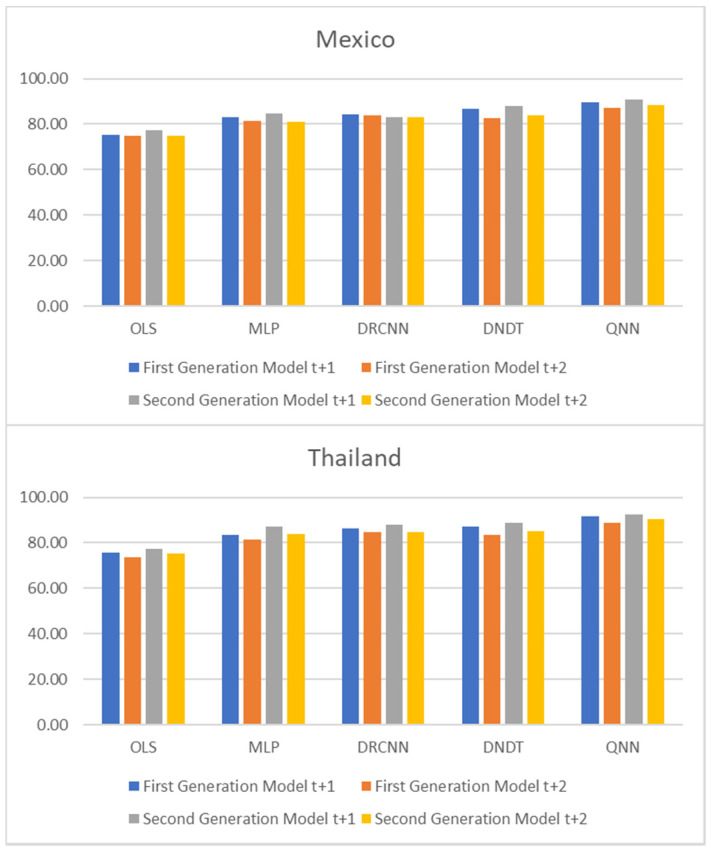
Multiple-step ahead forecasts in forecast horizon: accuracy.

**Figure 6:**
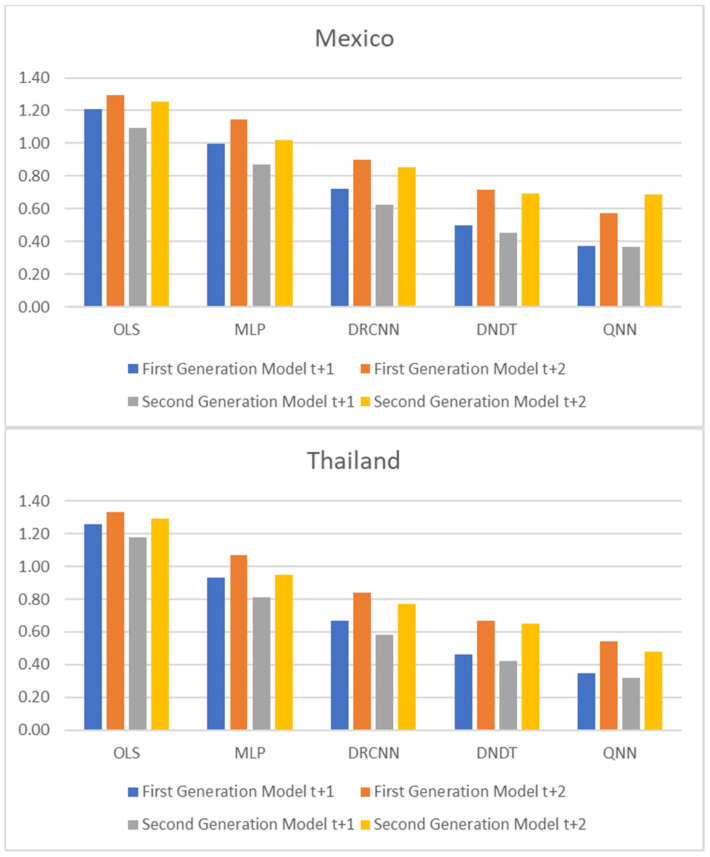
Multiple-step ahead forecasts in forecast horizon: RMSE.

**Figure 7:**
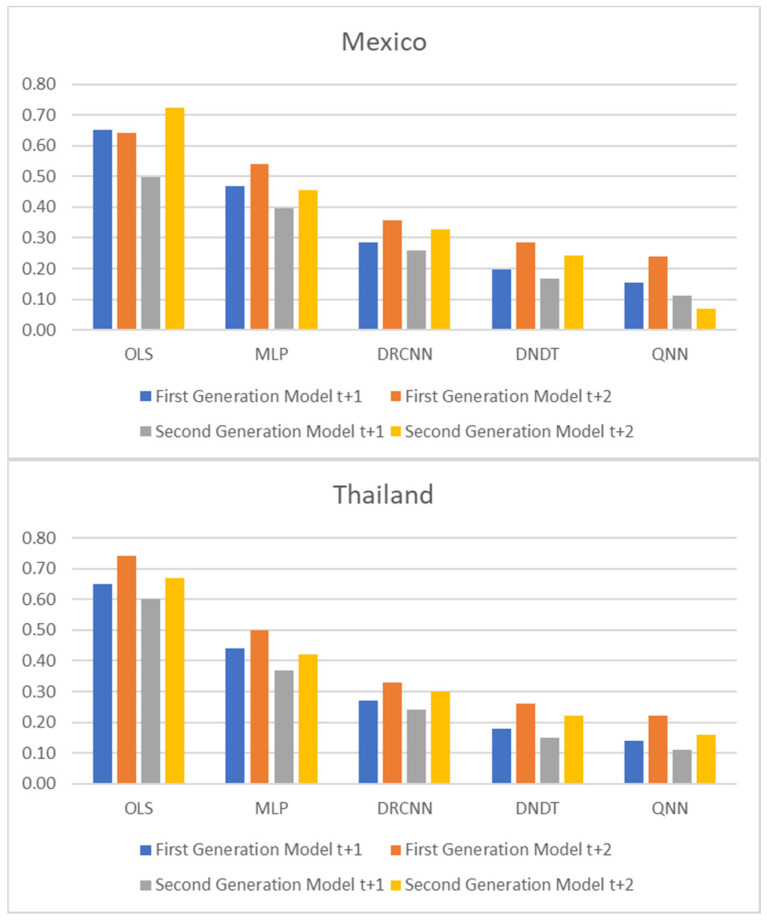
Multiple-step ahead forecasts in forecast horizon: MAPE.

To perform multiple-step-ahead prediction to obtain greater robustness of results, we use the iterative strategy. For this, we have trained the models for prediction for one step and two forward steps, that is, for the moments *t* + 1 and *t* + 2 [38]. These forecasted data for *t* + 1 and *t* + 2 are included in the data sample as actual observations. Table 4 and Table 5, and Figure 5, Figure 6 and Figure 7 point out the accuracy and residual results (RMSE and MAPE) for one-year and two-year forecasting horizons. For *t* + 1, the range of precision for the four neural networks techniques is 83.07–90.94% overall, being in the model of QNN where the percentage of accuracy is higher (90.94%) for the Mexican case. With the OLS method, the accuracy decreases to 74.72–74.90%. On the same line, for the Thai case, the precision range has been 83.34–92.63%, with QNN being again the methodology with the highest precision (92.63%). With the OLS method, the accuracy decreases to 75.64–77.15%. For *t* + 2, this range of precision is 81.34–89.52%, being also the method of QNN in which the percentage of accuracy is higher (89.52%) for the Mexican estimations. For the OLS method, the accuracy decreases to the range of 72.78–73.81%. Moreover, in *t* + 2 for the Thai estimations, again confirms the predictive superiority of QNN (90.54%). These results show the high precision and great robustness of the NN techniques.

## 6. Conclusions

This study has developed a new simulation of speculative attack models using machine learning techniques. Using data of period 1995–2019 for the cases of the currencies of Mexico and Thailand (Peso and Baht) and applying four different NN methods in the estimation of the first- and second-generation speculative attacks models to achieve a robust accuracy capacity, such as MLP, DRCNN, DNDT, and QNN. This last methodology is the one that has obtained the highest levels of precision. Most of the proposed NN methodologies have shown a low level of error and stability in the estimates made from speculative attack models, proving their interesting alternative to conventional statistical methods, such as OLS.

Besides, the target has been to improve the accuracy of previous studies using different methodologies. The results obtained in this research are higher than those obtained in the existing literature, with an accuracy range of 82.64–92.84% using the NN methods, while OLS method has only reached an accuracy range of 75.27–78.06%. It has also detected new significant variables to consider in speculative attacks models in weak currencies, allowing a high level of stability in the models developed over forecasting horizons of *t* + 1 and *t* + 2. In contrast to previous research, this study has been able to expand the estimation of speculative attacks in exchange rate attending to accuracy and error results. The results have identified a set of significant variables for each methodology applied and for each standard dependent variable. Furthermore, the time elapsed to make the estimates is less for the proposed NN techniques compared to the time needed for the OLS method. This makes an essential contribution to the field of computational macroeconomics and finance. The conclusions are relevant to public managers, financial analysts, central bankers, and other stakeholders in the foreign exchange markets, who are generally interested in knowing which indicators provide reliable, accurate, and potential forecasts of performance evolution. Our study suggests new explanatory significant variables to allow these agents to analyze the performance of speculative attack models. This research has also provided a new estimation analysis developed for speculative attacks using four NN methods, being the QNN the most accurate. Hence, this study attempts to contribute to existing knowledge in the field of machine learning. These new simulations of estimation can be used as a reference to improve decision-making in public and financial institutions.

In summary, this study provides a significant opportunity to contribute to the research line of currency crises and speculative attacks, since the results obtained have significant implications for the future decisions of public institutions, making it possible to avoid big negative changes of the trend of the exchange rate and the potential associated risks. It also helps these agents send warning signals to governments and central banks and avoid currency crisis losses derived from a huge decrease in the balance of payments. Further research could include speculative attack models with other new variables to take advantage of the benefits of machine learning techniques.

## Figures and Tables

**Table 1:** Results of accuracy evaluation: Mexico.

		First Generation Model		Second Generation Model	
		Training	Testing	Training	Testing
OLS	Accuracy (%)	78.45	75.27	80.02	77.41
	RMSE	1.12	1.20	1.01	1.10
	MAPE	0.57	0.61	0.41	0.47
MLP	Accuracy (%)	85.37	82.64	86.78	84.11
	RMSE	0.93	1.07	0.81	0.95
	MAPE	0.44	0.50	0.37	0.43
DRCNN	Accuracy (%)	90.04	84.30	91.95	86.18
	RMSE	0.67	0.84	0.59	0.80
	MAPE	0.27	0.33	0.24	0.31
DNDT	Accuracy (%)	92.15	88.10	93.62	89.05
	RMSE	0.46	0.67	0.42	0.65
	MAPE	0.18	0.27	0.16	0.23
QNN	Accuracy (%)	94.51	91.62	95.72	92.84
	RMSE	0.35	0.54	0.34	0.64
	MAPE	0.15	0.22	0.10	0.07

**Table 2:** Results of accuracy evaluation: Thailand.

		First Generation Model		Second Generation Model	
		Training	Testing	Training	Testing
OLS	Accuracy (%)	78.67	76.43	80.27	78.06
	RMSE	1.09	1.03	0.99	1.04
	MAPE	0.54	0.55	0.43	0.52
MLP	Accuracy (%)	87.81	85.01	89.27	86.52
	RMSE	0.87	1.00	0.76	0.89
	MAPE	0.41	0.47	0.34	0.40
DRCNN	Accuracy (%)	92.61	86.71	94.58	88.65
	RMSE	0.63	0.78	0.55	0.74
	MAPE	0.25	0.31	0.22	0.28
DNDT	Accuracy (%)	93.87	89.74	95.37	90.71
	RMSE	0.43	0.62	0.39	0.60
	MAPE	0.17	0.25	0.14	0.21
QNN	Accuracy (%)	96.27	93.32	97.50	94.57
	RMSE	0.32	0.50	0.32	0.60
	MAPE	0.13	0.21	0.10	0.06

**Table 3:** Comparison of testing results using Diebold-Mariano (DM) and Harvey-Leybourne-Newbold (HLN) tests.

	First Generation Model		Second Generation Model	
	**DM**	**HLN**	**DM**	**HLN**
OLS vs. MLP	−2.42 **	−2.31 *	−2.57 **	−2.25 **
OLS vs. DRCNN	−2.86 **	−2.57 **	−2.93 **	−2.83 **
OLS vs. DNDT	−3.02 **	−2.84 **	−2.99 **	−2.67 **
OLS vs. QNN	−3.17 **	−2.99 **	−3.29 **	−3.06 **
MLP vs. DRCNN	−2.15 **	−2.03 *	−2.47 *	−2.41 *
MLP vs. DNDT	−2.34 *	−2.17 **	−2.63 **	−2.49 **
MLP vs. QNN	−2.76 **	−2.62 **	−3.20 **	−3.07 **
DRCNN vs. DNDT	−2.08 *	−1.93 *	−2.47 *	−2.36 *
DRCNN vs. QNN	−2.53 *	−2.14 *	−2.45 **	−2.28 *
DNDT vs. QNN	−2.11 *	−1.97 *	−2.46 *	−2.13 **

* Indicates significance at the 5% level. ** Indicates significance at the 10% level.

**Table 4:** Multiple-step ahead forecasts in forecast horizon = *t* + 1 and *t* + 2 (Mexico).

		First Generation Model		Second Generation Model	
		*t* + 1	*t* + 2	*t* + 1	*t* + 2
OLS	Accuracy (%)	74.72	73.81	74.90	72.78
	RMSE	1.32	1.38	1.19	1.42
	MAPE	0.71	0.75	0.58	0.81
MLP	Accuracy (%)	83.07	81.34	84.51	80.89
	RMSE	1.00	1.15	0.87	1.02
	MAPE	0.47	0.54	0.40	0.46
DRCNN	Accuracy (%)	84.46	83.81	83.05	82.98
	RMSE	0.72	0.90	0.63	0.86
	MAPE	0.29	0.36	0.26	0.33
DNDT	Accuracy (%)	86.62	82.81	88.00	83.71
	RMSE	0.50	0.72	0.45	0.69
	MAPE	0.20	0.28	0.17	0.24
QNN	Accuracy (%)	89.78	87.04	90.94	89.52
	RMSE	0.37	0.58	0.37	0.53
	MAPE	0.16	0.24	0.11	0.15

**Table 5:** Multiple-step ahead forecasts in forecast horizon = *t* + 1 and *t* + 2 (Thailand).

		First Generation Model		Second Generation Model	
		*t* + 1	*t* + 2	*t* + 1	*t* + 2
OLS	Accuracy (%)	75.64	73.57	77.15	75.12
	RMSE	1.26	1.33	1.18	1.29
	MAPE	0.65	0.74	0.60	0.67
MLP	Accuracy (%)	83.34	81.58	87.16	83.94
	RMSE	0.93	1.07	0.81	0.95
	MAPE	0.44	0.50	0.37	0.42
DRCNN	Accuracy (%)	86.13	84.64	87.96	84.54
	RMSE	0.67	0.84	0.58	0.77
	MAPE	0.27	0.33	0.24	0.30
DNDT	Accuracy (%)	87.20	83.37	88.59	85.27
	RMSE	0.46	0.67	0.42	0.65
	MAPE	0.18	0.26	0.15	0.22
QNN	Accuracy (%)	91.45	88.66	92.63	90.54
	RMSE	0.35	0.54	0.32	0.48
	MAPE	0.14	0.22	0.11	0.14

## Data Availability

Data available on request due to restrictions.
